# Predictors of Methicillin-resistant *Staphylococcus aureus* infection in children with acute osteomyelitis

**DOI:** 10.1186/s13052-024-01780-0

**Published:** 2024-10-10

**Authors:** Kang Wang, Chen Wang, Hua Zhu, Yan Zou, Yanhua Feng, Fang Zhang, Yi Qu, Yiren Tian

**Affiliations:** 1 Department of Orthopedics, Children’s Hospital of Hebei Province, No. 133 Jianhua South Street, Shijiazhuang City, Hebei Province China; 2 Scientific Research Department, Children’s Hospital of Hebei Province, Shijiazhuang, Hebei China

**Keywords:** Acute hematogenous osteomyelitis, Pediatrics, Septic arthritis, *Panton–Valentine leucocidin*

## Abstract

**Background:**

This study aims to identify risk factors associated with Methicillin-resistant *Staphylococcus aureus* (MRSA) infection in children diagnosed with acute osteomyelitis (AO) and to elucidate the laboratory characteristics of these MRSA-infected children to enhance early targeted therapeutic interventions.

**Methods:**

We conducted a retrospective analysis involving 123 children with acute osteomyelitis treated at our hospital. Upon admission, we measured white blood cell (WBC) counts, C-reactive protein (CRP) levels, erythrocyte sedimentation rates (ESR), and platelet counts. Patients were categorized into two groups: the non-MRSA group (*n* = 73) and the MRSA group (*n* = 50), with values assigned as follows (non-MRSA group = 0, MRSA group = 1).

**Results:**

The MRSA group had a significantly higher average age compared to the non-MRSA group (*P* < 0.05). Notably, the incidence of suppurative arthritis was significantly lower in the MRSA group (*P* < 0.05). At the time of admission, CRP levels in the MRSA group were markedly elevated compared to those in the non-MRSA group (*P* < 0.01). After three days of empirical therapy, both WBC and CRP levels remained significantly higher in the MRSA group compared to the non-MRSA group (*P* < 0.05).

**Conclusions:**

In children newly admitted with acute osteomyelitis, a CRP level exceeding 73.23 µg/mL may indicate a high likelihood of MRSA infection. For children with AO who have been hospitalized for three days on empirical therapy, the presence of WBC > 10.95 × 10^9/L, CRP > 49.56 µg/mL, age > 3.5 years, and the absence of suppurative arthritis suggests a heightened risk of MRSA infection.

## Introduction

Osteomyelitis is an inflammatory condition of the bone resulting from microbial infection [1, 2]. In children, it presents unique challenges in both diagnosis and treatment [2]. Due to the often non-specific symptoms and systemic manifestations associated with pediatric cases, healthcare providers must maintain a high index of suspicion when evaluating children with limb pain. Early diagnosis and intervention are crucial, as delays can lead to severe complications, including growth arrest, limb deformities, cellulitis, abscess formation, septic arthritis, deep vein thrombosis, pathological fractures, sepsis, and even mortality [3–8]. 

*Staphylococcus aureus* is the predominant pathogen in childhood osteomyelitis [9], and hematogenous spread is the most common route of infection [10]. Among the various strains of *Staphylococcus aureus*, Methicillin-resistant *Staphylococcus aureus* (MRSA) has become a significant concern globally, associated with a wide range of hospital- and community-acquired infections, including bacteremia, respiratory tract infections, skin and soft tissue infections, osteomyelitis, and infectious arthritis [11]. The presence of MRSA biofilm in osteomyelitis poses additional treatment challenges, as such infections are notoriously difficult to eradicate and can be life-threatening [12]. 

Typically, the first-line antibiotics for osteomyelitis are beta-lactams, such as cefazolin, which are effective against Methicillin-sensitive *Staphylococcus aureus* (MSSA), Kingellakingae, Streptococcus pyogenes, and Streptococcus pneumoniae [13]. However, MRSA is a multidrug-resistant organism, rendering beta-lactam antibiotics ineffective. Upon confirmation of an MRSA infection, it is imperative to promptly transition to appropriate antibiotics, such as clindamycin or vancomycin [10]. 

In clinical practice, obtaining bacterial cultures from localized pus during surgery and performing sensitivity testing typically requires at least three days, while blood cultures along with sensitivity testing may take over five days, potentially delaying critical treatment. Therefore, this study aims to elucidate the characteristics of various early indicators in children with acute osteomyelitis infected with MRSA through multiple logistic regression analyses. Our goal is to assist clinicians in making timely decisions regarding the modification or escalation of antibiotic therapy.

## Patients and methods

### Patients

This retrospective study enrolled a total of 123 children with acute osteomyelitis (AO) who were hospitalized for surgical intervention at the Orthopedics Department of Hebei Children’s Hospital from January 2016 to December 2022. Patients were categorized into two groups based on the results of intraoperative bacterial cultures of pus: the non-MRSA group (*n* = 73) and the MRSA group (*n* = 50).

### Inclusion criteria

1. Children with AO admitted to the pediatric orthopedics department presenting with limb infections (symptoms persisting for less than 2 weeks, with affected limbs exhibiting swelling, pain, and restricted movement; X-ray and CT scans may be positive or negative upon admission; MRI findings revealed bone abnormalities, confirmed by intraoperative exploration).
2. Osteomyelitis diagnosis was established following empirical antibiotic therapy and surgical intervention.
3. Intraoperative bacterial cultures of pus were positive.
4. Routine blood tests, including C-reactive protein (CRP) and erythrocyte sedimentation rate (ESR), were conducted three days post-surgery.

### Exclusion criteria

1. Presence of other diseases in the affected limb, such as tumors or idiopathic arthritis.
2. Children with osteomyelitis without abscess formation who did not undergo surgical treatment.
3. Children presenting with chronic osteomyelitis upon admission (symptoms lasting more than two weeks with imaging evidence of bone destruction).

This study received approval from the Ethics Committee of Hebei Children’s Hospital (No. 203). The study protocol adhered to the Declaration of Helsinki, and written informed consent was obtained from the parents or guardians of all enrolled children.

### Treatment and indicators

We selected laboratory indicators that can be quickly assessed upon admission, with results available within four hours, including white blood cell count (WBC), CRP, ESR, platelet count, liver and kidney function tests, and coagulation profiles. Additionally, demographic data (gender, age) and clinical parameters (time from onset to hospital admission, presence of sepsis, and suppurative arthritis) were recorded. All patients received empirical treatment with cefazolin sodium for infection control. Once the patient’s condition stabilized, osteomyelitis fenestration/drainage surgery was performed, and pus samples were collected for bacterial culture and drug sensitivity testing post-operatively.

The diagnostic criteria for sepsis included clinical manifestations and any of the following: (1) Pathogenic bacteria cultured from blood or sterile body cavity; (2) A conditioned pathogen cultured from a blood specimen must also be identified in another blood sample or sterile site [14, 15]. In this study, sepsis diagnosis was based on bacteria cultured from blood samples. The criteria for pyogenic arthritis diagnosis were as follows: (1) Clinical symptoms such as joint or limb pain, limited range of motion, false paralysis, edema, erythema, fever, and tenderness; (2) Characteristic radiological or ultrasound findings; (3) Positive culture from bone or joint; (4) Aspiration of pus from joints or bones; (5) Relevant serological data, particularly WBC count, CRP levels, ESR, and blood cultures. Criterion 1 is essential, and at least two additional criteria must be met. In this study, pus was aspirated from joint punctures for the assessment of suppurative arthritis, leading to positive bacterial cultures [15]. Given that bacterial cultures typically require over three days for results, we supplemented the re-examination data at three days post-empirical treatment with WBC, CRP, ESR, and platelet counts to facilitate earlier assessment of potential MRSA infection, thus guiding antibiotic modification if necessary.

### Statistical analysis

Statistical analyses were performed using SPSS version 21.0 (IBM, USA). Data were categorized, and a binary classification database was established. Chi-square tests and T-tests were conducted initially, followed by determination of cutoff values for each dataset using ROC curves. Subsequently, univariate and multivariate logistic regression analyses were performed. A *p*-value of < 0.05 was considered statistically significant.

## Results

### Single factor analysis of MRSA-related factors

Chi-square tests and T-tests revealed that the age of children in the MRSA group was significantly greater than that in the non-MRSA group (*P* < 0.05). In contrast, the incidence of suppurative arthritis was significantly lower in the MRSA group compared to the non-MRSA group (*P* < 0.05). After three days of treatment, both white blood cell (WBC) counts and C-reactive protein (CRP) levels in the MRSA group were significantly elevated compared to the non-MRSA group (*P* < 0.05). Notably, CRP levels at admission were also significantly higher in the MRSA group (*P* < 0.01). No statistically significant differences were observed between the two groups regarding body mass index (BMI), history of preterm birth, breastfeeding status, infection mode, infection site, or other laboratory indicators (Table 1).

**Table 1:** Single factor analysis of MRSA related factors (Chi-square test and t-test)

Index	Non-MRSA (*n* = 73)	MRSA (*n* = 50)	t/X^2^	*P*
Age (year)	3.2 ± 3.7	5.1 ± 4.2	-2.543	0.013*
Gender/Male (*n*)	41	25	0.453	0.501
BMI<18 (*n*)	60	40	0.094	0.759
Premature delivery/yes (*n*)	5	0	3.570	0.059
Breast-feeding/no (*n*)	13	6	0.766	0.381
Infection mode/traumatic (*n*)	1	1	0.074	0.786
Infection location (left/right)/right (*n*)	45	29	0.164	0.685
Infection site (upper limb, lower limb)/ lower limb (*n*)	50	39	1.341	0.247
WBC at admission(/L)	16.59 ± 8.08	15.95 ± 8.32	0.423	0.673
WBC after 3 days of treatment(/L)	11.83 ± 4.72	14.04 ± 5.55	-2.377	0.019*
CRP at admission(ug/L)	67.03 ± 56.44	102.41 ± 68.59	-3.191	0.002**
CRPafter 3 days of treatment(ug/L)	20.88 ± 25.34	39.29 ± 48.17	-2.478	0.016*
ESR at admission(mm/h)	52.27 ± 23.54	47.40 ± 22.14	1.155	0.250
ESR after 3 days of treatment(mm/h)	38.99 ± 29.45	39.90 ± 26.62	-0.176	0.861
Plateletsat admission(/L)	431.62 ± 160.78	369.08 ± 194.09	1.946	0.054
Platelets after 3 days of treatment(/L)	524.01 ± 171.67	466.84 ± 197.81	1.705	0.091
Time from onset to admission > 7 days	21	10	1.210	0.271
FIB>5 g/L	30	27	1.987	0.159
APTT>30s	41	23	1.228	0.268
AST>40U/L	13	7	0.316	0.574
ALT >40U/L	11	6	0.235	0.628
Sepsis	41	31	0.416	0.519
Suppurative arthritis	42	19	4.530	0.033*

Note: *: *P* < 0.05, **: *P* < 0.01. #: Infection modes were divided into traumatic and hematogenous

**Fig. 1:**
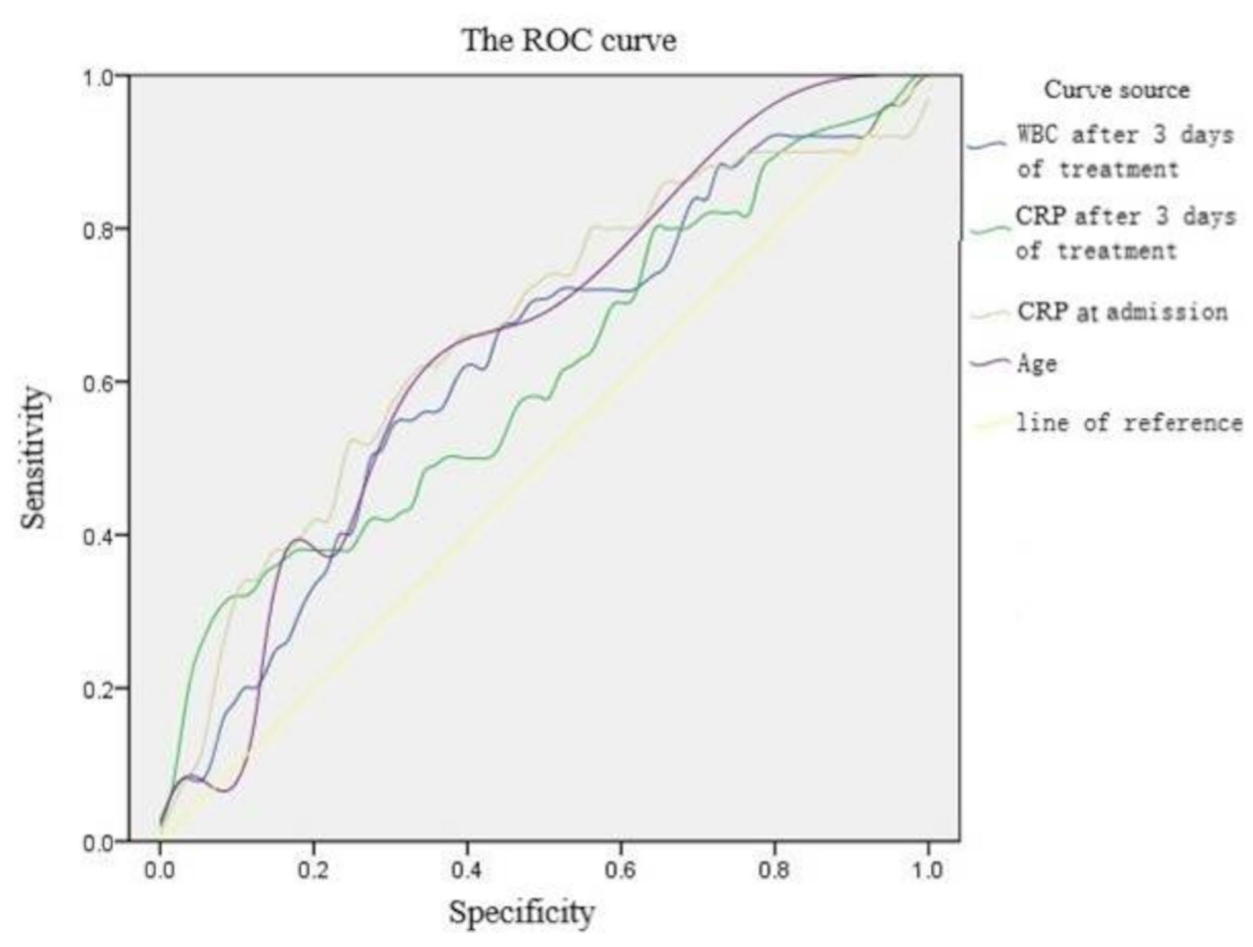
The ROC curve: WBC and CRP after 3 days of treatment, CRP at admission and age was considered statistically significant. The ROC curve is used to determine the critical value

Receiver Operating Characteristic (ROC) curve analysis was employed to determine cutoff values for various parameters. The cutoff values identified were as follows: age at 3.5 years, original CRP at 73.23 µg/mL, 3-day CRP at 49.56 µg/mL, and 3-day WBC count at 10.95 × 10^9/L. The ROC curve areas for platelet counts at admission and three days post-treatment were both less than 0.5, indicating no statistical significance (Fig. 1; Table 2).

**Table 2:** Area under the curve (AUC) of each value

Variables	AUC	standard error^a^	Asymptotic Sig.^b^	Asymptotic 95% confidence interval	
				Lower	Upper
WBC after 3 days of treatment	0.625	0.052	0.019	0.524	0.726
CRPafter 3 days of treatment	0.611	0.053	0.037	0.507	0.714
CRP at admission	0.663	0.051	0.002	0.563	0.763
Age	0.653	0.050	0.004	0.556	0.750

### Univariate logistic regression analysis of MRSA-related factors

Univariate logistic regression analysis indicated that age (OR: 2.683, 95% CI: 1.265–5.687, *P* = 0.010), absence of pyogenic arthritis (OR: 0.452, 95% CI: 0.217–0.944, *P* = 0.035), platelet count at admission (OR: 0.387, 95% CI: 0.171–0.875, *P* = 0.023), platelet count at three days post-treatment (OR: 0.387, 95% CI: 0.171–0.875, *P* = 0.023), CRP at admission (OR: 3.331, 95% CI: 1.571–7.062, *P* = 0.002), WBC count (OR: 2.723, 95% CI: 1.282–5.780, *P* = 0.009), and CRP after three days of treatment (OR: 5.255, 95% CI: 1.885–14.646, *P* = 0.002) were identified as independent risk factors for MRSA-related acute osteomyelitis (AO) in children (Table 3). These indicators were subsequently included in the multivariate analysis.

**Table 3:** Theunivariate Logistics regression analysis

Index	B	S.E	Wald	*P*	OR	OR (95%CI)
Age (>3.5 years = 1)	0.987	0.383	6.625	0.010*	2.683	1.265–5.687
Pyogenic arthritis (value = 1)	-0.793	0.375	4.464	0.035*	0.452	0.217–0.944
WBC after 3 days of treatment (>10.95*10^9^/L = 1)	1.002	0.384	6.799	0.009**	2.723	1.282–5.780
CRP at admission (> 73.23ug/mL = 1)	1.203	0.383	9.852	0.002**	3.331	1.571–7.062
CRPafter 3 days of treatment (>49.56 ug/mL = 1)	1.659	0.523	10.065	0.002**	5.255	1.885–14.646
Plateletatadmission (> 400*10^9^/L = 1)	-0.949	0.416	5.197	0.023*	0.387	0.171–0.875
Platelet after 3 days of treatment (> 400*10^9^/L = 1)	-0.949	0.416	5.197	0.023*	0.387	0.171–0.875

Note: *: compared with non-MRSA group, *P* < 0.05, **: compared with non-MRSA group, *P* < 0.01

### Multivariate logistic regression analysis of MRSA-related factors

The results of the multivariate logistic regression analysis are detailed in Table 4. In the analysis of admission indicators, CRP levels at admission emerged as a significant marker associated with MRSA infection (OR: 3.595, 95% CI: 1.617–7.995, *P* = 0.002). In the multivariate analysis of indicators measured three days post-treatment, significant associations with MRSA infection were observed for age (OR: 2.611, 95% CI: 1.034–6.597, *P* = 0.042), absence of pyogenic arthritis (OR: 0.345, 95% CI: 0.141–0.843, *P* = 0.020), CRP (OR: 3.741, 95% CI: 1.192–11.744, *P* = 0.024), and WBC count (OR: 5.091, 95% CI: 1.896–13.674, *P* = 0.001) (Table 5).

**Table 4:** The results ofmultivariate Logistics regression analysis of admission index

Index	B	S.E	Wald	*P*	OR	OR (95%CI)
Age (>3.5 years = 1)	0.728	0.432	2.846	0.092	2.072	0.889–4.830
Pyogenic arthritis (value = 1)	-0.628	0.411	2.333	0.127	0.534	0.238–1.195
CRP at admission (> 73.23ug/mL = 1)	1.280	0.408	9.847	0.002**	3.595	1.617–7.995
Plateletatadmission (> 400*10^9^/L = 1)	-0.727	0.469	2.403	0.121	0.483	0.193–1.212

Note: **: compared with non-MRSA group, *P* < 0.01

**Table 5:** The results ofmultivariate Logistics regression analysis of index after 3 days treatment

Index	B	S.E	Wald	*P*	OR	OR (95%CI)
Age (>3.5 years = 1)	0.960	0.473	4.122	0.042*	2.611	1.034–6.597
Pyogenic arthritis (value = 1)	-1.066	0.457	5.445	0.020*	0.345	0.141–0.843
WBC after 3 days of treatment (>10.95*10^9^/L = 1)	1.627	0.504	10.424	0.001**	5.091	1.896–13.674
CRPafter 3 days of treatment (>49.56 ug/mL = 1)	1.319	0.584	5.111	0.024*	3.741	1.192–11.744
Platelet after 3 days of treatment (> 400*10^9^/L = 1)	-0.825	0.510	2.618	0.106	0.438	0.161–1.191

Note: *: compared with non-MRSA group, *P* < 0.05, **: compared with non-MRSA group, *P* < 0.01

### Correlation analysis between surgical timing and laboratory indices

One-way analysis of variance assessing the timing of surgery revealed no significant effects on various laboratory indices, regardless of whether the surgery was performed within one day or three days of admission. Thus, no correlation was found between the timing of surgery and laboratory indicators (Tables 6 and 7).

**Table 6:** Univariate variance analysis of the effect of surgery within one day after admission on each indicator

Index	B	S.E	Wald	*P*	OR	OR (95%CI)
CRPafter 3 days of treatment (>49.56 ug/mL = 1)	0.302	0.494	0.374	0.541	1.352	0.514–3.560
WBC after 3 days of treatment (>10.95*10^9^/L = 1)	-0.060	0.391	0.023	0.879	0.942	0.438–2.027
Platelet after 3 days of treatment (> 400*10^9^/L = 1)	-0.154	0.435	0.126	0.723	0.857	0.365–2.011

Note: Surgery within one day after admission, time > 1 day is assigned as 1

**Table 7:** Univariate analysis of variance of the effect of surgery within three days after admission on each indicator

Index	B	S.E	Wald	*P*	OR	OR (95%CI)
CRPafter 3 days of treatment (>49.56 ug/mL = 1)	-0.299	0.803	0.139	0.710	0.742	0.154–3.578
WBC after 3 days of treatment (>10.95*10^9^/L = 1)	0.159	0.573	0.077	0.781	1.172	0.381–3.605
Platelet after 3 days of treatment (> 400*10^9^/L = 1)	0.331	0.686	0.233	0.629	1.392	0.363–5.339

Note: Surgery within three days after admission, time > 3 days is assigned as 1

## Discussion

Bone and joint infections in children are often severe, and misdiagnosis can lead to significant consequences, potentially impairing limb development and function, and even threatening life [16]. Rapid identification of osteomyelitis and appropriate surgical intervention, along with the judicious use of antibiotics, are crucial for effective treatment. Studies have demonstrated that, in the absence of surgical intervention, the administration of appropriate antibiotics alone can often yield satisfactory outcomes in treating acute osteomyelitis (AO) [17–19]. Consequently, the empirical selection of antibiotics must be guided by local epidemiology and clinical experience. In regions where the prevalence of methicillin-sensitive *Staphylococcus aureus* (MSSA) exceeds 90%, initial empiric antimicrobial therapy for neonatal AO should include beta-lactam antibiotics, such as anti-staphylococcal penicillins (e.g., oxacillin or nafcillin) or first-generation cephalosporins (e.g., cefazolin) [13]. Indeed, in our clinical practice, cefazolin is also the preferred antibiotic for treating AO in children. Unlike outpatient cases, hospitalized children with more severe AO typically require surgical intervention combined with targeted antibiotics. Indications for surgical treatment of AO include persistent symptoms unresponsive to empirical antibiotic therapy (e.g., fever, local inflammation), the presence of subperiosteal abscesses or other deep soft tissue infections (often associated with methicillin-resistant *Staphylococcus aureus* [MRSA] or virulence gene PVL-positive strains), concomitant septic arthritis—particularly in the hip and shoulder joints—necrotic bone, and sinus tract formation [18, 20, 21]. This study included the empirical treatment protocols experienced by children, encompassing both antibiotic therapy and surgical interventions (e.g., osteomyelitis debridement, decompression, irrigation, and drainage). In our clinical observations, the most common surgical indications for AO are subperiosteal abscess formation and septic arthritis. However, surgical treatment alone is insufficient, as it is often challenging to completely eradicate pathogenic bacteria during surgery, necessitating postoperative treatment with targeted antibiotics. Given that MRSA is resistant to commonly used empirical antibiotics and that bacterial cultures require time to yield results, early evidence-based antibiotic adjustments are critical.

Our primary aim was to determine, upon admission, whether children with AO were infected with MRSA, using various clinical indicators. Through t-tests, we found that children with MRSA had significantly higher C-reactive protein (CRP) levels at admission. After establishing a cutoff value using the receiver operating characteristic (ROC) curve, CRP emerged as the sole statistically significant indicator in multivariate analysis, with CRP levels > 73.23 µg/mL associated with a higher risk of MRSA infection. Roine et al. conducted a series of CRP and erythrocyte sedimentation rate (ESR) measurements in 63 children with acute hematogenous osteomyelitis, comparing clinical course and outcomes over 1 to 2 months. Their study found that high CRP values at admission (163 ± 108 mg/L) began to decline after the second day of treatment, and from day 4 onward, CRP values could distinguish between cases in remission and those with persistent symptoms at follow-up. Furthermore, children with imaging changes exhibited elevated CRP levels for a longer duration (32 ± 13 days). Although ESR was less predictive of clinical course, higher values on days 4–7 did distinguish symptomatic from asymptomatic children at follow-up. Roine et al. concluded that CRP monitoring could alert clinicians to complications and predict outcomes earlier than clinical symptoms or X-rays, with CRP being more valuable than ESR in this regard [22]. Similarly, our study found that CRP levels > 49.56 µg/mL after 3 days of empirical treatment indicated a poor response, likely due to MRSA infection, necessitating a change in antibiotics.

Previous studies have suggested that white blood cell (WBC) counts in children with AO are typically elevated, though not consistently [1]. In our study, WBC counts at admission did not differ significantly between groups. However, after 3 days of empirical treatment, significant differences were observed across all statistical methods. Using the ROC curve to determine the cutoff value, multivariate analysis revealed that WBC counts > 10.95 × 10^9/L after 3 days of treatment were associated with a higher risk of MRSA infection in children with AO. This threshold, slightly above the normal range, suggests that if the response to empirical treatment is effective, WBC counts should return to normal or near-normal levels within 3 days.

Bone and joint infections are generally considered more prevalent in children under 5 years of age [23–25]. Among the 123 children included in this study, the youngest was 21 days old, the oldest 15 years, with a mean age of 3.98 ± 4.0 years, consistent with previous studies. T-test results indicated that the mean age of MRSA-infected children was 5.08 years, significantly higher than the 3.22 years observed in non-MRSA children. In multivariate analysis after 3 days of empirical treatment, age > 3.5 years was identified as a risk factor for MRSA infection, suggesting that older children may be more susceptible to MRSA. We hypothesize that this may be due to the more robust immune response in older children, making them more susceptible to common bacteria, which are typically less virulent and easier to control with antibiotics, thereby preventing progression to osteomyelitis. However, as a multi-drug-resistant pathogen, MRSA is more challenging to control with commonly used cephalosporin antibiotics, leading to a higher incidence of osteomyelitis in older children. Additionally, septic arthritis may serve as a protective factor, as children with AO caused by MRSA are less likely to develop concomitant septic arthritis. There is currently limited research on the factors influencing the co-occurrence of osteomyelitis and septic arthritis. Furthermore, the time from symptom onset to hospital admission did not correlate with MRSA infection, suggesting that the timing of medical intervention did not significantly impact this outcome. However, this finding requires further investigation and validation. It is also important to note that our empirical treatment regimen included both antibiotic therapy and surgical intervention, with varying timing of surgery. Some children presented with severe symptoms, including early detection of pus via local puncture or MRI findings suggestive of abscess formation, leading to early surgical intervention. In contrast, children with milder symptoms or unclear diagnoses underwent surgery after a period of observation and treatment, resulting in delayed surgical intervention. To assess the impact of surgical timing on various laboratory indicators after 3 days of treatment, we conducted a one-way analysis of variance. The results indicated that whether surgery was performed within 1 day or within 3 days of admission, the timing of surgery did not affect laboratory indicators, and there was no correlation between surgical timing and laboratory results. This finding underscores the critical role of antibiotic therapy in managing AO.

Moreover, ESR is recognized as an important inflammatory marker, typically rising within 24–48 h after the onset of inflammation and gradually declining as the inflammation resolves [26]. However, ESR can be influenced by non-inflammatory factors such as age, anemia, pregnancy, medications, and obesity, which may lead to elevated values. In the study by Fincher RM et al., the sensitivity of ESR at 100 mm/h or more was low: 0.36 in patients with infections, 0.25 in patients with malignancies, and 0.21 in patients with non-infectious inflammatory diseases. Nevertheless, ESR specificity was high: 0.96 for malignancies, 0.97 for infections, and 0.99 for the “disease” index, with a positive predictive value (PPV) of 90% for identifying a significant increase in ESR [27]. In our study, ESR did not prove useful in determining MRSA infection at admission, and due to its slow decline, it remained statistically insignificant 3 days after empirical treatment.

However, this study has some limitations. First, the sample size is relatively small, with all patient data derived from a single hospital, potentially limiting the generalizability of our findings due to regional and treatment scope differences. Second, the study did not include sufficient indicators, such as imaging findings and detailed antibiotic classifications. Third, this paper does not analyze the interactions between multiple factors. In future research, we plan to conduct a multi-center study on pediatric orthopedic infectious diseases, expanding the sample size and incorporating additional indicators to further substantiate our conclusions.

## Conclusions

For newly admitted children with AO, a CRP level > 73.23 µg/mL suggests a high likelihood of MRSA infection. For children hospitalized with AO who have undergone 3 days of empirical therapy, a WBC count > 10.95 × 10^9/L, CRP level > 49.56 µg/mL, age > 3.5 years, and the absence of septic arthritis are associated with a higher risk of MRSA infection. These findings can guide clinicians in timely adjustment of antibiotics based on patient conditions.

## Data Availability

The datasets analyzed during the current study are not publicly available due to the personal privacy but are available from the corresponding author on reasonable request.

## Abbreviations

*MRSA*: Methicillin-resistant *Staphylococcus aureus*
AO: Acute osteomyelitis
WBC: White blood cell
CRP: C-reactive protein
ESR: Erythrocyte sedimentation rate
MSSA: Methicillin-sensitive *Staphylococcus aureus*
CRP: C-reactive protein
WBC: White blood cell
